# The Impact of COVID-19 in Reshaping Graduate Medical Education: Harnessing Hybrid Learning and Virtual Training

**DOI:** 10.7759/cureus.56790

**Published:** 2024-03-23

**Authors:** Abdualla Ben Ammer, Jennifer L Bryan, Ali Abbas Asghar-Ali

**Affiliations:** 1 Psychiatry and Behavioral Sciences, Baylor College of Medicine, Houston, USA; 2 South Central Mental Illness Research, Education and Clinical Center, Michael E. DeBakey Veterans Affairs Medical Center, Houston, USA; 3 Veterans Affairs Health Systems Research Center for Innovations in Quality, Effectiveness and Safety, Michael E. DeBakey Veterans Affairs Medical Center, Houston, USA

**Keywords:** didactics, clinical rounds, telehealth, residency education, covid-19 pandemic

## Abstract

Introduction

At the start of the COVID-19 pandemic, many graduate medical education (GME) programs switched from in-person to virtual training to ensure a safe learning environment. However, the preferences of US residents in the wake of the COVID-19 pandemic are largely unknown.

Objective

The authors surveyed PGY-2 psychiatry residents about their perception of the pandemic's impact on their clinical skills, didactics experience, training preferences, and future career perceptions.

Methods

The cross-sectional study was conducted from October 31, 2021, to December 31, 2021. The authors emailed a survey to directors of US general psychiatry residency programs to disseminate to PGY-2 residents. The survey had Likert-scale and open-ended questions about the pandemic's perceived impact on PGY-1 training and future training preferences. The authors used descriptive statistics for Likert-scale questions and reflexive thematic analysis for open-ended questions.

Results

Out of an estimated 1800 residents, only 116 (6.4%) participated; post-pandemic preferences emerged. A strong preference was expressed for hybrid didactics, combining in-person and virtual learning. Virtual patient evaluations, especially in emergency and inpatient settings, were highly valued. Conversely, entirely virtual didactics and clinical rounds were deemed least preferred, emphasizing the importance of interactive, hands-on learning experiences.

Conclusions

Respondents emphasized the significance of incorporating hybrid models for both in-patient care and didactic sessions in GME. These preferences signify the need for adaptable and flexible approaches to education in psychiatry residency programs as we emerge from the pandemic.

## Introduction

The arrival of the COVID-19 pandemic in the United States necessitated rapid adaptations in residency training programs [1,2]. Adaptations implemented during the initial months of the pandemic, including the transition to virtual didactic sessions and virtual clinical care, had significant consequences and led to decreased caseloads, rising utilization of telehealth services, and reduced social interactions among residents and supervisors. As a result, certain residents encountered declining patient caseloads, heightened anxiety regarding their professional futures, and a perception that their training had been compromised [3-9].

Limited research has investigated the impact of the COVID-19 pandemic on psychiatry residency training in the United States, with a national survey highlighting decreased in-person rotations, varied changes in call hour duties, and concerns about burnout among residents, while knowledge gaps remain regarding alterations in learning structures, the impact on PGY-1 residents, and preferences for post-pandemic training [7]. Addressing the aftermath of the public health emergency is crucial for graduate medical education (GME), involving comprehensive assessments to understand specialty-specific impacts and learning gaps, as well as implementing strategies such as mental health support, hybrid learning models, telehealth training, and professional development opportunities. The research seeks to investigate the impact of the COVID-19 pandemic on PGY-1 psychiatry residents' training experiences, encompassing clinical, didactic, and career aspects.

## Materials and methods

The cross-sectional study was conducted between October 31, 2021, and December 31, 2021. It was electronically sent to directors of psychiatry residency programs with a request to forward the email to their PGY-2 residents. This list of psychiatry programs was obtained from the FREIDA™, the American Medical Association Residency & Fellowship Database®, which is publicly available. Following the initial invitation email, two follow-up emails were sent. Of 284 program directors contacted, five emails were undeliverable, three directors were no longer serving, and three directors stated that they had new programs without eligible PGY-2 residents. The survey was anonymous, and the only demographic information collected was the state where residents completed their PGY-1 residency training. The investigators did not have any means of identifying participants based on their state. Participants were not compensated, and no incentives were provided to participate. The study included first-year psychiatry residents who completed their first year in the academic year 2020-2021 and excluded senior psychiatry residents and residents from other specialties.

The survey, created by the authors, included five subtopics: general questions, clinical skills, didactic learning, preferences for future education, and psychiatric careers. It started with qualifying questions that asked whether participants completed PGY-1 psychiatry training in the academic year 2020-2021 and whether they were PGY-2 psychiatry residents at the time of completing the survey and asked which state their PGY-1 program was in. This was followed by questions on a Likert scale. Participants were asked about the impact of the COVID-19 pandemic on their education on a Likert scale from 1 (not at all) to 5 (a great deal), how they felt the training program adjusted to meet their educational needs during the first year of the COVID-19 pandemic on a Likert scale from 1 (not well at all) to 5 (very well), and how often they used telehealth on a Likert scale from 1 (never) to 5 (always). The Likert-scale questions for the remainder of the survey were asked on a scale from 1 (strongly disagree) to 5 (strongly agree).

Four open-ended questions were asked: “In what way(s) did the pandemic 'positively' impact your clinical learning experience?,” “In what way(s) did the pandemic negatively impact your clinical learning experience?,” “In what way(s) did the pandemic positively impact your didactic learning experience?,” and “In what way(s) did the pandemic or negatively impact your didactic learning experience?”

Descriptive statistics were calculated using IBM SPSS version 27 (SPSS Inc., Chicago, IL, USA). Open-ended questions were reviewed, based on thematic analysis. Author JB reviewed the quotes from each question and highlighted themes that emerged. The team selected quotes that were most impactful from each theme. Example quotes were selected to give insight to qualitative results. This study was approved by the Baylor College of Medicine Institutional Review Board.

## Results

Of the estimated 1800 PGY-2 psychiatry residents across the nation, 116 (6.4%) participated in the survey. Respondents represented 28 states, with most being from Texas (n=28, 24.1%; see Table 1 for the full list of states). Table 2 presents the means and standard deviations for the survey questions.

**Table 1:** Number and percentage of respondents by state Total number of respondents (N)=116

State	N	Percent
Alabama	2	1.72%
Alaska	0	0.00%
Arizona	2	1.72%
Arkansas	0	0.00%
California	4	3.45%
Colorado	4	3.45%
Connecticut	1	0.86%
Delaware	0	0.00%
Florida	3	2.59%
Georgia	0	0.00%
Hawaii	0	0.00%
Idaho	0	0.00%
Illinois	3	2.59%
Indiana	0	0.00%
Iowa	0	0.00%
Kansas	1	0.86%
Kentucky	2	1.72%
Louisiana	0	0.00%
Maine	0	0.00%
Maryland	1	0.86%
Massachusetts	4	3.45%
Michigan	3	2.59%
Minnesota	6	5.17%
Mississippi	2	1.72%
Missouri	3	2.59%
Montana	0	0.00%
Nebraska	0	0.00%
Nevada	1	0.86%
New Hampshire	2	1.72%
New Jersey	3	2.59%
New Mexico	0	0.00%
New York	14	12.07%
North Carolina	6	5.17%
North Dakota	0	0.00%
Ohio	2	1.72%
Oklahoma	0	0.00%
Oregon	2	1.72%
Pennsylvania	6	5.17%
Rhode Island	0	0.00%
South Carolina	1	0.86%
South Dakota	0	0.00%
Tennessee	0	0.00%
Texas	28	24.14%
Utah	0	0.00%
Vermont	0	0.00%
Virginia	3	2.59%
Washington	0	0.00%
West Virginia	1	0.86%
Wisconsin	6	5.17%
Wyoming	0	0.00%

**Table 2:** Descriptive statistics for survey questions *Item responses options are 1 = none at all, 2 = a little, 3= a moderate amount, 4= a lot, 5 = a great deal. **Item response options are 1 = no well at all, 2 = not well, 3 = somewhat well, 4 = well, 5 = very well. ***Item response options are 1 = never, 2 = rarely, 3 = sometimes 4 = usually, 5 = always. The remaining item response options are 1 = strongly disagree, 2 = disagree, 3 = neither agree or disagree, 4 = agree, 5 = strongly agree.

	Response 1	Response 2	Response 3	Response 4	Response 5	Mean	Standard deviation
General impact questions							
To what extent do you feel the COVID-19 pandemic impacted your education?*	4(3.45%)	19(16.38%)	40(34.48%)	33(28.45%)	20(17.24%)	3.4	1.06
How well do you feel your program adjusted to the pandemic during your PGY-1 psychiatry training to meet your educational needs?**	0(0.00%)	12(10.34%)	42(36.21%)	48(41.38%)	14(12.07%)	3.55	0.84
Over the course of the PGY-1, on average how often did you see patients via telehealth?***	27(23.28%)	38(32.76%)	39(33.62%)	10(8.62%)	2(1.72%)	2.33	0.98
Clinical training							
I felt less sure about my diagnosis of patients I saw via tele health	7(6.19%)	35(30.97%)	35(30.97%)	31(27.43%)	5(4.42%)	2.93	1.01
My supervisor provided enough time for teaching	7(6.03%)	11(9.48%)	17(14.66%)	65(56.03%)	16(13.79%)	3.62	1.04
I acquired tele-medicine as a new skill	11(9.65%)	11(9.65%)	25(21.93%)	52(45.61%)	15(13.16%)	3.43	1.14
I saw enough patients to meet my clinical training needs	1(0.87%)	5(4.35%)	10(8.70%)	57(49.57%)	42(36.52%)	4.17	0.83
I was able to gain insight from my peers during virtual clinical rounds	9(8.04%)	17(15.18%)	41(36.61%)	39(34.82%)	6(5.36%)	3.14	1.01
I have reached the milestones that are expected of a PGY-1 psychiatry resident	2(1.74%)	1(0.87%)	9(7.83%)	67(58.26%)	36(31.30%)	4.17	0.75
Didactic learning							
I felt engaged with instructors	17(14.66%)	46(39.66%)	13(11.21%)	38(32.76%)	2(1.72%)	2.67	1.13
Instructors adapted to teaching in a virtual environment	6(5.17%)	16(13.79%)	13(11.21%)	75(64.66%)	6(5.17%)	3.51	0.97
I learned from my peers during virtual sessions	8(6.90%)	22(18.97%)	21(18.10%)	61(52.59%)	4(3.45%)	3.27	1.03
Virtual learning met my educational needs	11(9.48%)	28(24.14%)	26(22.41%)	44(37.93%)	7(6.03%)	3.07	1.12
Preferences for future education							
A combination of virtual and in-person didactics	7(6.03%)	13(11.21%)	14(12.07%)	55(47.41%)	27(23.28%)	3.71	1.13
Completely virtual didactics	38(33.04%)	33(28.70%)	18(15.65%)	16(13.91%)	10(8.70%)	2.37	1.31
Have the option of evaluating patients virtually in ER/inpatient settings	20(17.39%)	12(10.43%)	19(16.52%)	51(44.35%)	13(11.30%)	3.22	1.29
A combination of virtual and in-person clinical rounds	23(20.00%)	33(28.70%)	16(13.91%)	37(32.17%)	6(5.22%)	2.74	1.25
Completely virtual clinical rounds	56(48.70%)	35(30.43%)	18(15.65%)	2(1.74%)	4(3.48%)	1.81	1
Psychiatric career							
My skills as a psychiatrist were irreversibly compromised due to my PGY-1 training during the pandemic	58(50.00%)	46(39.66%)	10(8.62%)	2(1.72%)	0(0.00%)	1.62	0.72
My PGY-1 training during a pandemic has better equipped me to respond to public health emergencies	4(3.45%)	14(12.07%)	24(20.69%)	65(56.03%)	9(7.76%)	3.53	0.93
I am more likely to look for job opportunities with a virtual component due to my experiences during my PGY-1 training	10(8.62%)	19(16.38%)	33(28.45%)	38(32.76%)	16(13.79%)	3.27	1.15

General impact

Respondents reported that the COVID-19 pandemic moderately impacted their training but that their respective training programs responded well (54.3% reported well or very well). Most reported infrequently caring for patients via telehealth (59.1% reported never or rarely). Although the open-ended questions did not specifically inquire, many residents spontaneously mentioned the impact on their well-being and community during their PGY-1 training. Some reported a sense of unity with others, while others reported negative impacts on their connections with others and increased stress. An example quote: "We spend 4 months on inpatient medicine, often caring for ICU patients. Because so many people were dying, it created a sense of ’we’re in this together’ that helped us get through it and brought us closer with our co-residents across disciplines. I found that attendings were more understanding." However, others reported negative impacts to connection with others and increased stress. An example quote: "So many patients died, and it was really depressing and sad. No one else in our program except PGY-1s are on inpatient medicine. Most of the psych faculty who do outpatient work would say things that were totally out of touch and, like, check your privilege, talking to us about how sad and lonely they were sitting at home doing telehealth all day. Well, ok, great, we’re in the trenches, putting ourselves and our families at risk dealing with multiple patients dying every day. I had nightmares about chest X-ray." Another resident highlighted how stressful their first-year experience was: "It added an additional level of stress to a notoriously stressful first year. The burden of fearing for you or your loved ones becoming ill due to your job was traumatic. It felt like residents had to take the burden of care from other providers with the same amount of pay and benefits. With that combination of issues, residents had to work significantly harder to meet their clinical expectations while managing mental and physical health."

Clinical training

 Residents reported varying experiences with their clinical training; however, most reported enough clinical experiences (86.2% agreed or strongly agreed) to reach expected milestones (89.7% agreed or strongly agreed). In the open-ended questions both increased and reduced patient caseloads were reported. As an example, a resident said, “Working at lower capacity allowed for greater educational time and individualized teaching from attendings”; while others reported being overwhelmed by the patient load, as shown in the following quote: “The majority of the year was spent on medicine treating covid patients in the medicine wards and intensive care unit. It made for a very challenging time. Volume was insane.” Additionally, some residents reported training in more patient care settings; while others reported fewer opportunities.

Didactic experience

 Respondents noted instructors adapted well (70.7% agreed or strongly agreed) to a virtual environment, and they learned from their peer residents (56.9% agreed or strongly agreed). However, they were less likely to report feeling engaged with the instructor (18.1% agreed or strongly agreed). In the open-ended questions respondents reported several positives of virtual didactics, including that those didactics were easier to attend, Zoom was engaging, and they enjoyed guest speakers from other institutions; and there was a positive impact on resident wellness. As an example, a resident stated, “Having virtual didactics allowed for improved attendance to didactics. If I was running late due to patient care, I could always join on my phone to continue learning rather than completely miss part of lecture.” However, other residents reported negative experiences with virtual didactics, such as technology issues, “Zoom fatigue,” and topics that did not translate well to a virtual environment; many times learners would be distracted by clinical responsibilities during didactics and not be able to connect with their peers. As an example, a resident stated, “Most people continued working during the lecture/taking calls in the background rather than paying attention to the lecture. This once protected time for learning is now just extra time to work.”

Preferences for future training

Respondents mostly preferred a combination of virtual and in-person didactics (70.7% agreed or strongly agreed) and the option of providing virtual patient care in emergency department/inpatient settings (56.0% agreed or strongly agreed). The least preferred option was completely virtual clinical rounds (6.0% agreed or strongly agreed) and completely virtual didactics (23.3% agreed or strongly disagreed). 

Career as a future psychiatrist

Respondents reported they were somewhat more likely to seek a job with a virtual component (47.4% agreed or strongly agreed) and they felt better equipped to respond to public health emergencies (64.7% agreed or strongly agreed). Only two respondents (1% agreed or strongly agreed) said their training during the pandemic had irreversibly compromised their skills as a psychiatrist.

## Discussion

Before the COVID-19 pandemic, resident education mostly involved in-person didactics and clinical rounds [2]. Some residents reported virtual didactics benefited their well-being, while others reported zoom fatigue and felt didactic time was no longer protected learning time and clinical work overtook that time. Despite rapid changes, many residents perceived that they were still able to meet expected milestones and that their careers as psychiatrists were not irreversibly compromised.

These findings may be generalized to medical residency training beyond psychiatry. As pandemic restrictions have decreased, it may be beneficial for residency programs to adopt a hybrid learning structure. Hybrid didactics can offer various advantages, such as allowing residents to join sessions even if they are delayed by patient care needs and enabling external guest lectures. By incorporating the flexibility of virtual learning with face-to-face learning, programs can create a more dynamic learning environment that caters to the diverse needs of residents. Moreover, providing telehealth services can meet patients’ preferences.

The COVID-19 pandemic has undeniably reshaped the landscape of medical education, with significant implications for residency training across various specialties, including pathology, surgery, radiology, orthopedics, and emergency medicine. Each specialty faced unique challenges and made adaptations in response to the pandemic, highlighting the common thread of a shift toward virtual learning, decreased clinical exposure, and concerns regarding resident well-being and educational outcomes [10-16].

This study had several limitations. Given the low response rate and generally positive findings, it is possible those with a more negative experience did not participate. The study was self-report, among residents who had no previous experience in postgraduate training, which may have impacted their perceptions. Future research could investigate how PGY-1 and other residents viewed the pandemic’s impact on their training at a later date and in conjunction with preceptor evaluations.

## Conclusions

This study emphasizes the impacts of the COVID-19 pandemic on residents' education, particularly during their first year of training. Respondents frequently noted the expansion of telehealth in patient care, didactics, and clinical learning. They also perceived that their learning milestones were met and that their careers were not irreversibly impacted, despite the sudden shift to a virtual learning environment. Going forward, as residency programs move beyond pandemic restrictions, they might consider adopting hybrid learning models that integrate the flexibility of virtual learning with face-to-face learning, especially for hands-on exams. Additionally, participants emphasized the need for GME to investigate the expansion of virtual training for residents, particularly in the use of telehealth for patient care and educational activities.
